# Cell-Free DNA: An Underestimated Source of Antibiotic Resistance Gene Dissemination at the Interface Between Human Activities and Downstream Environments in the Context of Wastewater Reuse

**DOI:** 10.3389/fmicb.2020.00671

**Published:** 2020-04-21

**Authors:** Markus Woegerbauer, Xavier Bellanger, Christophe Merlin

**Affiliations:** ^1^Department for Integrative Risk Assessment, Division for Risk Assessment, Data and Statistics, AGES – Austrian Agency for Health and Food Safety, Vienna, Austria; ^2^Université de Lorraine, CNRS, LCPME, Nancy, France

**Keywords:** wastewater reuse, antibiotic resistance, horizontal gene transfer, free DNA, transduced DNA, membrane vesicles associated DNA

## Abstract

The dissemination of antimicrobial resistance (AMR) is one of the biggest challenges faced by mankind in the public health domains. It is currently favored by a lack of confinement between waste disposal and food production in the environmental compartment. To date, much effort has been devoted into the elucidation and control of cell-associated propagation of AMR. However, substantial knowledge gaps remain on the contribution of cell-free DNA to promote horizontal transfers of resistance genes in wastewater and downstream environments. Cell free DNA, which covers free extracellular DNA (exDNA) as well as DNA encapsulated in vesicles or bacteriophages, can persist after disinfection and promote gene transfer in the absence of physical and temporal contact between a donor and recipient bacteria. The increasing water scarcity associated to climatic change requires developing innovative wastewater reuse practices and, concomitantly, a robust evaluation of AMR occurrence by implementing treatment technologies able to exert a stringent control on AMR propagation in downstream environments exposed to treated or non-treated wastewater. This necessarily implies understanding the fate of ARGs on various forms of cell-free DNA, especially during treatment processes that are permissive to their formation. We propose that comprehensive approaches, investigating both the occurrence of ARGs and their compartmentalization in different forms of cellular or cell-free associated DNA should be established for each treatment technology. This should then allow selecting and tuning technologies for their capacity to limit the propagation of ARGs in any of their forms.

## Introduction: Colliding Challenges in Health and Sustainable Development

With 10 millions expected deaths per year by 2050, the mortality due to antimicrobial resistance (AMR) will surpass all known human diseases, making the fight against AMR one of the biggest challenges faced by Mankind in order to preserve the effectiveness of our modern medicine (de Kraker et al., 2016; O’Neill, 2016; Nature Microbiology supplement, 2016^1^). The emergence and dissemination of AMR is an unavoidable aspect of bacterial evolution following the consumption of antibiotics (Courvalin, 2005), but the excessive and inappropriate use of antimicrobials, together with the lack of innovation in discovering new antimicrobials, is driving modern medicine to the edge of a new dark age resembling the pre-antibiotic era (Levy and Marshall, 2004; Roberts and Simpson, 2008). This has led national and international organizations to take action in the public health and veterinary/farming domains for a better and confined usage of antimicrobials. But, are the proposed antibiotic stewardship and action plans sufficient for controlling the global increase of AMR? Surely not, as the spread of resistant bacteria (ARB) and their resistance genes (ARGs) over ecosystem boundaries appears now to be the dominant contributing factor for AMR dissemination (Collignon et al., 2018). Antimicrobial therapies exert selective processes allowing ARB to proliferate in microbiotas. Considering the continuum between human activities and the environment, AMR-determinants are also continuously released in anthropogenically-impacted environments where ARB can persist, accumulate, transfer their ARGs to indigenous microbes, and finally re-enter the food chain and be transferred to human and animal commensals and pathogens (Davies and Davies, 2010). Facing the global population growth, the environment is increasingly exposed to pollutants including ARB and ARGs. Concomitantly, the shortage in resources promotes an increasing demand for reuse and especially for recycled water. If water scarcity is another of the big challenges faced by humanity, the increasing need for wastewater reuse may also promote shortcuts in the AMR dissemination routes with bigger risks for human and animal health. Wastewater treatment plant (WWTP) effluents are at the interface between human activities and the downstream environment, therefore tackling water scarcity through water reuse will necessarily imply a better understanding of the AMR dissemination mechanisms to control them properly. Limiting the discharge of ARB in the downstream environment is obviously a critical point but this may not be enough to prevent the spread of ARGs toward and among environmentally adapted bacteria that may become reservoirs of resistances. Indeed, if controlling the discharge of ARB should provide a mean to limit the spread of ARGs involving cell-contact mechanism (e.g., conjugation), it just forgets to consider non-cell associated ARG transfer. As a matter of fact, non-cell associated ARG transfer is largely underestimated and we propose that cell-free sources of DNA are adequately considered for the risk assessment of ARG dissemination.

## Cell-Free DNA as a Neglected Source of Args

**FIGURE 1:**
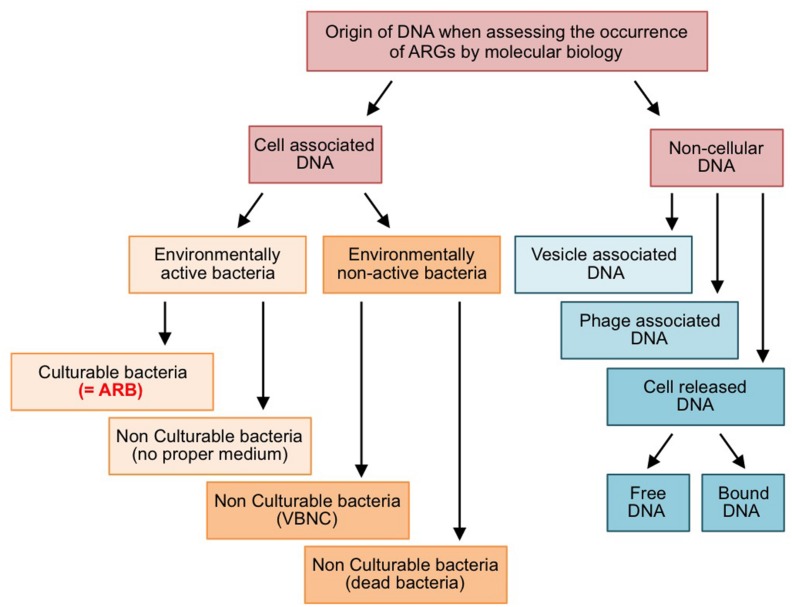
Forms and origins of ARGs quantified by molecular biology approaches. VBNC: viable but non-culturable bacteria.

The dissemination of ARB and ARGs in effluent-impacted environments is of concern when considering the safety aspects of reclaimed water (Hong et al., 2018). The efficiency of the processes implemented for treating effluents has been assessed by various techniques relying either on the culturability of ARB or on molecular approaches to estimate the abundance of ARGs. As a matter of fact, both approaches are not equivalent, because ARGs are not always associated with cultivable ARB (Figure 1). On the one hand, cell-associated ARGs can reside in bacteria that are non-cultivable (lack of proper culture medium, “viable but non-cultivable state,” or dead cells). On the other hand, there is also a non-negligible amount of ARGs carried on cell-free DNA (i.e., exDNA, bacteriophage-associated DNA, and membrane vesicle-associated DNA). This raises two interconnected questions with respect to wastewater reuse and associated wastewater treatment processes: (i) Is reclaimed wastewater adequately safe if a treatment process eliminates ARB below detection limits while ARGs are still present and quantifiable in the effluent? The correct answer will depend to some extent on the ability of ARB for regrowth, but undoubtedly also on the fate of ARGs encoded on cell-free DNA. Consequently, the second question arises: (ii) what is the persistence of ARGs associated to cell-free DNAs and their probability to be transferred and expressed in living bacteria? In the following sections, three main mechanisms leading to cell-free DNAs and their uptake by living bacteria, namely natural transformation of bacteria with free exDNA, transduction by bacteriophages and related particles, and DNA transfer by membrane vesicles (MVs), will be discussed in the context of the horizontal gene transfer (HGT) of ARGs. At present it is believed that, in the environment, HGT is mostly driven by conjugative elements, which explains why they have focused much attention so far (Sørensen et al., 2005). However, this lack of interest for alternative HGT pathways involving cell-free DNA probably reflects the technical difficulties to study such pathway rather than a real estimation of their implication. Actually, in spite of contributing to the global occurrence of ARGs in the environment, cell-free DNAs may well represent a significant source for ARG dissemination that suffers a lack of attention although it should be considered as a hazard in the context of the wastewater reuse.

## Membrane Vesicle-Mediated Horizontal Gene Transfer

Lately, the formation of MVs by bacteria has received increasing attention, as they seem to be involved in the transport of a myriad of biomolecules between bacteria (Grüll et al., 2018; Toyofuku et al., 2019). Several types of MVs can be distinguished. In Gram-negative bacteria, budding of the outer membrane (OM) results in the formation of 10–300 nm wide OM vesicles (OMVs) trapping periplasmic material. Several non-exclusive mechanisms promoting local OM shape alterations have been proposed to explain OMV formation (Gaudin et al., 2013; Toyofuku et al., 2015; Roier et al., 2016). It starts with a loss of linkage between the OM and peptidoglycan, which then allows OM budding to happen. This is further promoted by various factors affecting the curvature of the membranes such as the accumulation of OM proteins, the turgor pressure, the OM rigidity due to the accumulation saturated fatty acids, the anionic charge repulsion between saccharide chains of lipopolysaccharides (LPS), the wrong localization of phospholipids in the outer layer of the OM (instead of LPS), and the insertion of amphiphilic molecules in the OM (e.g., quinolones). Sometimes, plasma membranes are dragged along during OM budding, thus leading to double layered outer-inner membrane vesicles (O-IMVs) with cytoplasmic content, including DNA (Pérez-Cruz et al., 2015). MVs are also produced by Gram-positive bacteria following localized cell wall damages with the concomitant action of the turgor pressure (Toyofuku et al., 2017), and were also observed in Archaea (Gaudin et al., 2013). The size and the relative amount of MVs tend to increase in antimicrobial-stressed environments which may favor OM destabilization and budding (Fulsundar et al., 2014; Devos et al., 2017).

Several important functions have been attributed to bacterial MVs (Toyofuku et al., 2015). MVs appear as a major component of biofilm matrices and they may be involved in bacterial cell protection by adsorbing antimicrobial agents, including bacteriophages (Schooling and Beveridge, 2006). MVs also seem to fulfill multiple roles depending on the kind of biological material they trap and transport: cell-cell communication (transport of signaling molecules), virulence (transport of toxins), electron transfer (transport of redox-active proteins), and finally HGT (transport of DNA) (Toyofuku et al., 2017; Grüll et al., 2018), for which the size distribution of MV-packaged DNA ranges from a few base pairs to several dozen kilobases depending on the species and its physiological state (Klieve et al., 2005; Biller et al., 2017; Bitto et al., 2017). Several studies reported MV-assisted transformation of chromosomal and plasmid DNA, between either Gram-negative bacteria, Gram-positive bacteria or Archaea, and even from bacteria to the nuclear fraction of eukaryotic cells (Bitto et al., 2017). In 2011, Rumbo et al. (2011) reported the MV-assisted transfer of the plasmid-borne *blaOXA24* resistance gene from two clinical isolates of *Acinetobacter baumannii* into the susceptible strain ATCC 17978, therefore demonstrating that ARGs are technically transferable by MV. However, demonstration of MV-mediated HGT remains relatively sporadic and solely assayed in pure cultures, where both the environmental dimension and frequency of the phenomenon remain unexplored (Domingues and Nielsen, 2017).

The global occurrence of ARGs in environmental MVs is a subject that is largely unexplored, and even less is known regarding MVs in WWTP effluents or in effluent-impacted downstream environments. This probably comes from the technical challenges of separating MVs from bacteriophages as they share common properties for isolation (Biller et al., 2017), raising the question of the accuracy of what was once attributed to bacteriophages. However, it can be taken for granted that (i) MVs are produced by numerous bacteria, (ii) they can transport any kind of DNA and this should include ARGs, (iii) with a membrane fusion-based mechanisms for MV-assisted DNA transformation, no taxonomic limitation are expected regarding the DNA uptake process, (iv) maintaining the acquired DNA in bacterial cell is likely to meet the same limitations as for natural transformation (see below), and (v) the budding process is favored in antibiotic-stress environments. Putting these observations together, the implication of MVs in the dissemination of ARGs in WWTPs and downstream environments is likely but its incidence remains to be elucidated. With this respect, structures once known as “biological colloids” or “liposomes-like biogenic materials,” probably MVs, have already been described in wastewater effluents and sometime reported to affect the operation and the quality of treatment processes (Song et al., 2010; Barry et al., 2014; Toyofuku et al., 2015). Second, considering that WWTP combines both MV forming bacteria and stressing molecules (e.g., antibiotics), it is of prime importance to determine: (i) whether the induction of MV formation can occur during the wastewater treatment processes, (ii) where it occurs during the process, (iii) whether a given advanced treatment process could eliminate or retain MVs in the final effluents, and finally (iv) the fate of the MVs discharged from WWTPs in downstream (aquatic and terrestrial) environments.

## Horizontal Gene Transfer by Natural Transformation

Natural transformation describes the physiologically regulated uptake and genomic integration of free exDNA by competent bacteria (Nielsen and Van Elsas, 2019). Approximately 130 bacterial species from almost all branches of the bacterial tree of life, including important human and animal pathogens and soil bacteria, have been characterized as being able to develop competence under naturally occurring environmental conditions (Johnston et al., 2014; Woegerbauer et al., 2015). The DNA uptake process either utilizes distinct species-specific uptake sequences or is sequence-independent (Nielsen and Van Elsas, 2019). Efficient transformation depends on sequence similarity between the incoming linear DNA and the recipient genome for homologous recombination (Lorenz and Wackernagel, 1994). The efficiency of recombination decreases in a log-linear relationship with increasing sequence divergence between DNA partner molecules (Ray et al., 2009), dropping below the detection limit if sequence identity is lower than ca. 75% (Townsend et al., 2003). Two additional recombination processes concerning bacterial transformation, homology facilitated illegitimate recombination (HFIR; de Vries and Wackernagel, 2002), and short patch double illegitimate recombination (SPDIR; Harms et al., 2016), with relaxed sequence identity requirements, have been identified but are supposed to occur at insignificant rates in natural bacterial populations (Nielsen and Van Elsas, 2019). However, for the formation of mosaic ARGs, HFIR is thought to play a decisive role (Woegerbauer et al., 2015). Natural transformation does not require physical contact between donor and recipient bacteria but allows large tempo-spatial separation between the genetic source of information and the recipient (Johnston et al., 2014).

Antibiotic resistance genes are prevalent in WWTPs (Reinthaler et al., 2003; da Costa et al., 2006; Auerbach et al., 2007; Novo and Manaia, 2010; Munir et al., 2011; Yuan et al., 2014) and in their effluents (Liu et al., 2018). Initially attributed to intracellular DNA, it was demonstrated that ARGs encoded on free exDNA persist throughout different WWTP compartments (1–16 ng/ml) and ARG-associated exDNA is present in substantial amounts even in purified discharged effluents (Zhang et al., 2018). Although WWTPs reduce ARG loads by up to 3 orders of magnitude (Auerbach et al., 2007; Zhang et al., 2009; Munir et al., 2011), 10^5^–10^9^ copies of ARGs per liter (total DNA) of effluent are generally discharged (Gao et al., 2012; Zhang et al., 2018), which can foster the spread of ARGs in receiving environments (Proia et al., 2016). Upon entry into the wastewater environment, a fraction of exDNA adsorbs to particulate material, rendering it inaccessible to nuclease (Breazeal et al., 2013). A rapid degradation of DNA from external sources (e.g., from decomposing plant leaves or from spikes with purified plasmid DNA) is observed in non-sterile soils (Romanowski et al., 1992; Pote et al., 2005). A similar fate of exDNA originating from WWTP effluents is expected if reclaimed water is used for irrigation of agricultural soils. However, a small fraction of the inoculated exDNA is usually adsorbed to sand particles, clay and other soil colloids (Nielsen et al., 2007). Some DNA is also tightly bound to humic acids (Crecchio and Stotzky, 1998). These interactions stabilize dsDNA fragments in soil ecosystems and render them inaccessible for DNases (Nielsen et al., 2007). ExDNA fragments have therefore been reported to be detectable for up to 2 years in agricultural soil environments (Gebhard and Smalla, 1999; Nielsen et al., 2007). Although bound exDNA remains to be accessible for uptake by competent bacteria, the observed long-term physical persistence of DNA fragments appears to be in contrast to a shorter-term availability of exDNA for transformation of competent bacteria as observed in soil microcosm experiments (Nielsen et al., 2007). Actively excreted exDNA is a core structural component of bacterial biofilms especially in activated sludge (Dominiak et al., 2011). High abundances of extracellular ARGs (1.7 × 10^3^–4.2 × 10^8^ copies per gram of dry weight) have been observed in sludge (Zhang et al., 2013). Exposure to sub-inhibitory concentrations of antibiotics leads to increased ARG concentrations and higher amounts of exDNA in the exposed ecosystem (Johnson et al., 2013; Lewenza, 2013; Doroshenko et al., 2014; Hathroubi et al., 2015; Schilcher et al., 2016), thus making exDNA an important source of genetic information fueling HGT by natural transformation (Wang et al., 2002; Merod and Wuertz, 2014).

While natural transformation in wastewater environments is believed to occur, informations on transformation frequencies, and conditions that may induce competence of bacteria in these habitats are scarce. Agricultural soils and associated crop plants are prominent downstream environments exposed to ARGs encoded on exDNA if irrigated with treated WWTP effluents. A limiting factor for efficient transformation is the availability of free exDNA (Nielsen and Van Elsas, 2019). However, the situation is unclear as fragments of exDNA show long-term persistence (several years) in soil and other natural environments (Overballe-Petersen et al., 2013), and despite adsorption to soil particles exDNA is capable to transform competent cells in these environments (Romanowski et al., 1993; Thomas and Nielsen, 2005; Morrissey et al., 2015). HGT by natural bacterial transformation in soils is assumed as an infrequent process (Nielsen et al., 1998; Pietramellara et al., 2006; Pietramellara et al., 2007). However, this may be an underestimation due to the substantial number of non-cultivable soil bacteria that may be able to develop competence (Pietramellara et al., 2009).

Environmental conditions in soil adverse to bacterial growth and proliferation are expected to be the rule rather than the exception. HGT via transformation, however, may occur in the absence of host cell proliferation. There is still a substantial lack of thorough understanding of the real, microhabitat-specific, impact of these processes in relation to the selective forces that operate in these ecological niches. Bioinformatics analyses of soil metagenomes provide strong evidence for the importance of HGT for bacterial biodiversity in natural soil habitats. It must be stressed that the impact and long-term effects of natural transformation – which is considered a rare event in soil environments – is not determined by the frequency of the event but by selection (Nielsen and Townsend, 2004; Townsend et al., 2012; Nielsen et al., 2014). Stochastic survival, dissemination and natural selection will determine the outcome of natural transformation (Nielsen and Van Elsas, 2019).

## Bacteriophage-Assisted DNA Transfer

Transduction is an HGT mechanism involving bacterial viruses, i.e., bacteriophages (or phages) that represent the most abundant class of microorganisms on Earth (10^30^–10^32^ phages *vs*. 10^29^–10^31^ bacteria; Chibani-Chennoufi et al., 2004; Brabban et al., 2005). After their injection into a bacterial cell, phage genomes can either enter into an active replicative state, leading to the production of new phages (lytic state), or into a dormancy state (lysogenic state). The latter case is restricted to temperate bacteriophages, which integrate into the host genome as a prophage. Classically, there are two distinct mechanisms of transduction. The “generalized transduction,” mediated by either lytic or temperate (lysogenic) phages, allows transferring any part of a bacterial genome form one bacteria to another, by accidentally packaging bacterial DNA fragments instead of the phage genome. On the other hand, the “specialized transduction,” solely mediated by temperate phages, leads to the HGT of the few bacterial genes located in the vicinity of the prophage insertion sites in the bacterial genome. Here, the inaccurate excision of prophage genome leads to the formation of chimerical prophage-bacterial DNA molecules that are packaged in the newly formed virions. When virions contain bacterial DNA, they retain their “nucleic acid syringe” function, allowing the packaged bacterial DNA to be injected into a new recipient bacterium (Calendar, 2012). A successful gene transduction event requires the injected DNA, or part of it, to integrate into the bacterial host genome to be maintained and expressed. Linear bacterial DNA fragments can be integrated in the new host genome thanks to its homologous or illegitimate recombination machineries, whereas transduced mobile genetic elements (MGEs) such as transposons or plasmids can ensure their maintenance by transposition or self-replication, respectively (Berg et al., 1983; Jiang and Paul, 1998; Calendar, 2012). It is worth noting that gene transfer can also be mediated by phage-resembling particles encoded by bacterial chromosomes, called Gene Transfer Agents (GTAs; Grüll et al., 2018). Lately, a new phenomenon called “lateral transduction,” initially described in *Staphylococcus aureus* prophages, seems to be involved in gene transfers in a wide range of bacterial species (Chen et al., 2018; Chiang et al., 2019). Briefly, upon entry in lytic state, some prophages do not start their activation by an excision from the chromosome but undergo an *in situ* genome replication. Then, DNA packaging begins while the prophages are still integrated in the chromosome, and may proceeds beyond the prophage boundaries, using over hundreds kilobases of adjacent chromosomal DNA as a substrate for filling capsids. The gene transfer frequencies based on lateral transduction appeared to be higher than those corresponding to generalized or specialized transductions.

Although HGT mediated by infectious phages and GTAs have been well studied *in vitro*, their occurrence in natural environments remains to be fully elucidated (Balcázar, 2018; Grüll et al., 2018). Metagenomic or qPCR-based studies investigating the content of phages/GTAs purified from natural environments impacted by human activities, animal/Human feces, or WWTP sludge and effluents have shown that both particles often carried ARGs (Muniesa et al., 2013; Calero-Cáceres et al., 2014; Ross and Topp, 2015; Colombo et al., 2016; Lekunberri et al., 2017; Brown-Jaque et al., 2018; Larrañaga et al., 2018; Wang et al., 2018). A recent work based on database mining has shown that phage genomes rarely encode ARGs by themselves, therefore suggesting that most phages/GTAs associated ARGs are on transduced bacterial DNA fragments (Enault et al., 2017). Although important for maintaining the transduced DNA in the new host, to date, the association of such transduced ARGs with MGEs has only be described recently (Yang et al., 2018).

Many environmental stressors, anthropogenic or not, have been demonstrated to induce phage proliferation within environmental microbial communities. These stressors are often of chemical nature: genotoxic compounds (e.g., antibiotics), metallic trace elements (e.g., Cu^2+^, Cd^2+^), polycyclic aromatic hydrocarbons (e.g., phenanthrene, pyrene), and polychlorinated biphenyls or pesticides (Danovaro et al., 2003; Jończyk et al., 2011; Lee et al., 2006; Allen et al., 2010); but can also be of physico-chemical nature such as temperature increase, UV radiation, and Ca^2+^ depletion or inorganic phosphate enrichment (Danovaro et al., 2003; Yue et al., 2012; Colomer-Lluch et al., 2014). These stressors are commonly encountered in WWTPs, as pollutants or as disinfection processes, and in the environments in which treated/untreated wastewater are discharged. Therefore, studying the ARG content of phages/GTAs released from wastewater treatment facilities is of particular interest, to identify specific pollutants and processes favoring the formation of transducing particles, and evaluating the role of phages/GTAs in the dissemination of ARGs in the downstream environments (Calero-Cáceres et al., 2014).

Considering the environmental abundance of phages/GTAs and their *in vitro* ability to mobilize ARGs, they are assumed to play a significant role in the environmental dissemination of ARGs/MGEs as suggest by a few studies, especially in environments impacted by human activities (Kenzaka et al., 2007; Ross and Topp, 2015; Shousha et al., 2015). Unfortunately, the effective transfer of a specific ARG by transduction has never been formally shown in environmental settings (Ross and Topp, 2015). Consequently, the real chances of success of the transduction of ARGs, i.e., their stable integration into the genome of a recipient bacterium after being injected into the cell, have never been explored and the global propensity of the phenomenon to occur in the environment remains to be assessed quantitatively. The phage host range is believed to be one of the limiting parameters for transduction to occur. However, recent works in this field tend to show that phages exhibiting broad host ranges, sometime spanning multiple genera, are more common than anticipated (de Jonge et al., 2019). At this stage, it is worth distinguishing between the “penetrative-host-range,” which refers to the bacteria into which phages can deliver DNA (or RNA) without necessarily engendering a progeny, and the narrower “productive-host-range,” i.e., the bacteria in which infections effectively lead to the production and release of virions (Hyman and Abedon, 2010). Consequently, transducing phages can deliver DNA in larger host-ranges than their usually reported “productive-host-range.” Altogether, the global transducing properties of natural phage communities remain to be determined but, as recently suggested, broad-host-range transducing phages are likely frequent in the environment (Kenzaka et al., 2007; de Jonge et al., 2019).

## Are Wastewater Treatment Effluents Hot Spots for Hgt Promoted by Cell-Free DNA?

**FIGURE 2:**
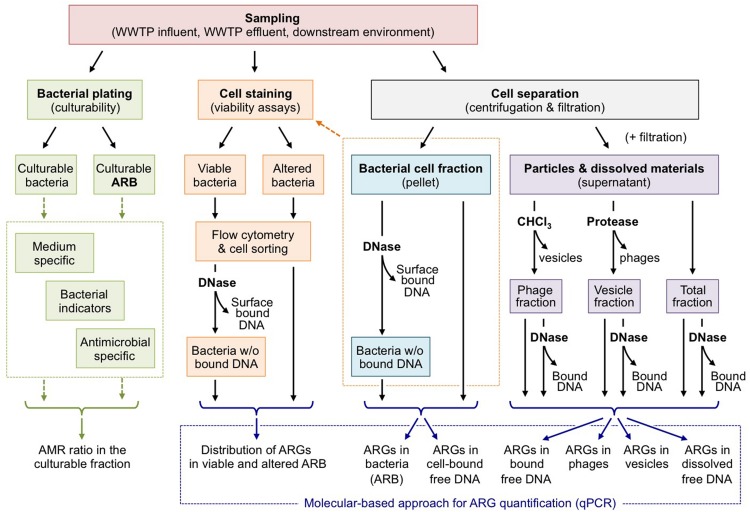
Assessing the distribution of ARGs in the different compartments of wastewater effluents. As stated by Biller et al. (2017) or Soler et al. (2015) MVs and phages remain difficult to separate. This figure should be understood as a working hypothesis.

Nowadays, it is well understood that HGT plays a significant role in the dissemination of ARGs in bacterial communities. However, to date much effort has primarily focused on elucidating HGT events promoted by MGEs, especially conjugative elements, which rely on direct cell-to-cell contacts (Sørensen et al., 2005). Actually, if conjugation is perceived as the key mechanism of HGT in soil (van Elsas et al., 2003; Heuer and Smalla, 2012, Zhang et al., 2014), there is currently no information available on the relative importance of transformation compared to conjugation and transduction (Nielsen and Van Elsas, 2019). Yet, HGT can also occur by alternative mechanisms comprising transduction, transformation and membrane vesicle-assisted transformation, for which the transfer of DNA can take place distantly from the donor host in time and space. This is particularly worrying in the context of wastewater reuse because conventional estimation of effluent quality based on the reduction of ARB will be of no help to estimate the occurrence of ARGs in free exDNA (solvated or associated to particles), and/or phage/vesicle fractions. The abundance of ARGs in all of these fractions tends to increase in anthropogenically stressed environments. This is also the case for WWTPs, which receive both ARB and antibiotics that have been reported to increase vesicle formation or phage production. At present, there is an obvious lack of empirical evidence assessing the type and extent of cell-free DNAs in the environment and their persistence in various WWTP processes. This results from the fact that most emphasis has been given to HGT by “conventional” MGEs and little case has been given to HGT by cell-free DNA. Considering the non-negligible probability of ARG dissemination by means of cell-free DNAs, there is an urgent need to assess both the persistence of such cell-free DNAs and the efficiency of the cognate HGT mechanisms along wastewater reuse processes, to limit the dissemination of ARGs and preserve the downstream environments (i.e., food chain and water cycles) from being exposed. Such assessment will necessarily require implementing an integrated approach to determine the distribution of ARGs in each biological compartment (free DNA, cell-, phage-, or vesicle-associated DNA), as exemplified in Figure 2. The critical point of such approach lies on the separation of each biological compartment before quantifying their ARG abundances using standard molecular methods. Knowing the occurrence of relevant ARGs in each biological compartment and estimating independently their respective HGT frequencies, will improve ARG risk assessment in the reuse context and, in the meantime, allow adapting processes and practices to target ARGs in specific biological compartments. To date, we miss a comprehensive and integrated approach comparing the relative contribution of conjugation, transformation, and phage-/vesicle-assisted transduction in the HGT of relevant ARGs, so as to identify the most effective mechanisms. Such approach necessarily implies microcosm-/reactor-based experiments implementing sensitive molecular strategies for the detection of so-called “rare events” in complex environmental matrices, as proposed by Bellanger et al. (2014) for conjugation, for instance. Next to the efficiency of the different transfer mechanisms, the persistence of the different biological entities (DNA, phages, and vesicles) is a key parameter that needs to be further evaluated in the environmental matrices considered. With this respect, it is worth mentioning that a recent work considering raw wastewater shown that ARGs in phages tend to be more resistant than those in bacteria to temperature and pH stresses, as well as UV irradiation or chlorination disinfection processes (Calero-Cáceres and Muniesa, 2016). This longer persistence of ARGs in phages has also been demonstrated in the downstream environment (Calero-Cáceres et al., 2017). In wastewater, Zhang et al. (2018) reported persistence of exDNA and observed a substantial increase of the abundance ratio of exDNA to internal DNA along the whole WWTP treatment process indicative for an amplification of ARGs encoded on free exDNA during wastewater treatment. Disinfection measures appear to increase the content of extracellular ARGs in WWTPs and corresponding effluents (Zheng et al., 2017; Liu et al., 2018; Yuan et al., 2019). A rapid degradation of DNA from external sources (e.g., from decomposing plant leaves or from spikes with purified plasmid DNA) is observed in non-sterile soils (Romanowski et al., 1992; Pote et al., 2005). A similar fate of exDNA originating from WWTP effluents is expected if reclaimed water is used for irrigation of agricultural soils. However, a small fraction of the inoculated exDNA is usually adsorbed to sand particles, clay and other soil colloids (Nielsen et al., 2007). Some DNA is also tightly bound to humic acids (Crecchio and Stotzky, 1998). These interactions stabilize dsDNA fragments in soil ecosystems and render them inaccessible for DNases (Nielsen et al., 2007). ExDNA fragments have therefore been reported to be detectable for up to 2 years in agricultural soil environments (Gebhard and Smalla, 1999; Nielsen et al., 2007). To the best of our knowledge, the persistence of MV in environmental matrices remains to be elucidated. Knowing the persistence of phages, vesicles and naked DNA, as well as their propensity to promote gene transfer will certainly be a step forward for assessing risk associated to ARG propagation, even if relatively rare. However, it should be kept in mind that ARG propagation is an intricate combination of DNA transfer, selection and clonal expansion of fit organisms. This means that a rare transfer event may become significant when considering the full scale of the receiving environment or the volumes of wastewater processed in WWTPs. Under such circumstances, even extremely rare transfer events may be rapidly amplified and disseminated in exposed bacterial populations. In summary, we propose to give more attention to cell-free transfer of ARGs that has been underestimated so far. Phages, vesicles and naked DNA share in common to possibly promote ARG transfer between bacteria that are distant in space and time. This rises the question of the efficiency of disinfection processes to control ARG propagation, since they solely inactivate ARB, while (i) alternative biological supports of ARGs may pass between cracks, (ii) some cell stresses may promote the release of DNA, phage and vesicles. Putting back these observations into the water reuse context, special care should be given to the identification of the ARG biological supports released in WWTP effluents so as to adapt the treatment processes consequently.

## Author Contributions

MW, XB, and CM equally contributed to this manuscript under the supervision of CM.

## Conflict of Interest

The authors declare that the research was conducted in the absence of any commercial or financial relationships that could be construed as a potential conflict of interest.

## Footnotes

1 Resistance ascends the political summit. Nat. Microbiol. 1:16223; Davies, S. C. (2011). Annual Report of the Chief Medical Officer, Vol. 2, Infections and the Rise of Antimicrobial Resistance (Department of Health, 2013) (http://go.nature.com/2ecdeKA); Report to the President on Combating Antibiotic Resistance (President’s Council of Advisors on Science and Technology, 2014) (https://www.cdc.gov/drugresistance/pdf/report-to-the-president-on-combating-antibiotic-resistance.pdf); Global Action Plan on Antimicrobial Resistance (WHO, 2015) (http://go.nature.com/2bbijap); A Scientific Roadmap for Antibiotic Discovery (The Pew Charitable Trusts, 2016) (http://go.nature.com/2eofNsf); Tackling Drug-Resistant Infections Globally: Final Report and Recommendations (Review on Antimicrobial Resistance, 2016) (http://go.nature.com/2e3bkMJ); Draft Political Declaration of the High-level Meeting of the General Assembly on Antimicrobial Resistance (UN, 2016) (http://go.nature.com/2e3bMdF); CARA: The conscience of antimicrobial resistance accountability The Center For Disease Dynamics, Economics & Policy (2016) (http://go.nature.com/2dGFhQv).
